# Unlocking the Secrets of Human Milk: Isolation and Characterization of Extracellular Vesicles

**DOI:** 10.1016/j.advnut.2025.100430

**Published:** 2025-04-25

**Authors:** Klaudia Tiszbein, Izabela Koss-Mikołajczyk, Dorota Martysiak-Żurowska

**Affiliations:** Faculty of Chemistry, Gdańsk University of Technology, Poland

**Keywords:** extracellular vesicles, ultracentrifugation, flow cytometry, exosomes, human milk, breast milk

## Abstract

Extracellular vesicles from human milk (HMEVs) are crucial for neonatal development, immune modulation, and protection against pathogens. However, the lack of standardized isolation and characterization protocols poses significant challenges. This review aims to evaluate and compare various methods for the isolation and characterization of HMEVs, highlighting their effectiveness and potential applications. Preliminary purification steps, including the removal of cells, fat globules, and casein micelles, enhance the purity of isolated HMEVs. We categorized isolation methods into density-based, size-based, and affinity-based techniques. Density-based methods include differential and density gradient ultracentrifugation. Size-based methods encompass polymer precipitation, membrane filtration, electrophoretic filtration, size exclusion chromatography, and microfluidics. Affinity-based methods involve immunoisolation using antibodies specific to HMEV surface proteins. Characterization techniques discussed include flow cytometry, dynamic light scattering, nanoparticle tracking analysis, tunable resistive pulse sensing, electron microscopy, atomic force microscopy, confocal microscopy, western blotting, ELISA, and lateral flow immunoassay systems. Differential ultracentrifugation, considered the “gold standard,” provides high purity but is time-consuming. Density gradient ultracentrifugation offers precise separation. Size-based methods like polyethylene glycol precipitation and membrane filtration are simple and fast. Electrophoretic filtration and microfluidics provide precise control of sample flow. Affinity-based methods are highly specific but costly. Advanced characterization techniques provide comprehensive insights into HMEV properties and functions. Standardizing isolation protocols and employing advanced characterization techniques are essential for advancing HMEV research. Future studies should focus on understanding the molecular mechanisms of HMEVs, exploring the impact of maternal health, and developing targeted delivery technologies. These efforts will enhance the therapeutic potential of HMEVs in neonatal care and contribute to personalized nutritional interventions.

**Statements of significance**

This review aims to evaluate and compare various methods for the isolation and characterization of human milk extracellular vesicles, highlighting their effectiveness and potential applications. It also emphasizes the importance of preliminary purification of human milk in order to remove cells, fat globules, and casein micelles, the natural components of human milk, that, if not removed, may be confused with extracellular vesicles during later analysis.

## Introduction

Human milk (HM) contains perfectly balanced nutrients necessary for optimal growth and development of an infant [1]. Additionally, HM compounds boost the infant’s immune system thanks to the presence of antibodies and other immune-supporting components [2]. Therefore, HM, with its complex composition tailored to meet the evolving nutritional needs of infants, has gained significant scientific interest in recent years.

Despite extensive research on the composition of HM, data on HM extracellular vesicles (EV) (HMEVs) is surprisingly scarce. These small, membrane-bound structures contain molecules such as proteins, lipids, and nucleic acids [3]. It is believed that HMEVs may have a profound impact on the health and development of infants, yet comprehensive research elucidating their exact contribution is lacking because of, among others, nonoptimized methods of isolation and characterization.

The International Society for Extracellular Vesicles, to improve rigor, reproducibility, and transparency in EVs research, provided minimal information for studies of EVs (MISEV2023). MISEV2023 highlights the importance of reporting pre-analytical variables—such as donor characteristics, sample collection, and storage methods — as well as distinguishing EVs from nonvesicular extracellular particles, which often co-isolate and may confound results. Rather than prescribing fixed protocols, MISEV2023 offers a flexible framework applicable across diverse sample types and research settings. It supports best practices in EV isolation, characterization, and functional studies, including in translational and clinical contexts [4].

This review aims to compare various methods currently used for the isolation and characterization of HMEVs. Each proposed method will be evaluated for efficacy, reliability, and potential to be used in the context of HMEVs. By synthesizing existing knowledge and exploring diverse methodologies, this review seeks to contribute to the progress of investigations on the HMEVs.

## HMEVs

HMEVs are predominantly produced and secreted by mammary gland epithelial cells during lactation [5,6]. Other cells present in HM, such as lymphocytes, macrophages, and stem cells, may also contribute to the EVs found in milk. Additionally, EVs from other organ cells can enter the milk *via* systemic circulation, further enriching the EV pool in HM [[7], [8], [9]]. HMEVs are a heterogeneous group of structures consisting of 3 main subpopulations: exosomes, microvesicles, and apoptotic bodies, which differ in their biogenesis mechanisms, composition, and functions [10].

Exosomes are vesicles measuring 40–150 nm, formed through the inward budding of early endosome membranes, leading to the creation of multivesicular bodies [[11], [12], [13]]. Molecular cargo, including proteins, lipids, and nucleic acids, is sorted and directed to forming exosomes *via* mechanisms dependent on the endosomal sorting complex required for transport (ESCRT) [10]. There is also an ESCRT-independent pathway, where sphingomyelin is converted into ceramide, promoting the formation of lipid domains conducive to vesicle formation [14]. Once multivesicular bodies are fully formed, they can fuse with the plasma membrane, releasing exosomes into the extracellular space, where they participate in intercellular communication [10,15].

Microvesicles are larger than exosomes (diameter 100–1000 nm) and formed by the direct outward budding of the plasma membrane [[16], [17], [18], [19]]. Their formation is regulated by changes in membrane asymmetry and the cytoskeleton. Calcium-dependent enzymes like flippase and floppase redistribute phospholipids between the inner and outer membrane layers, leading to destabilization and microvesicle formation [10,20]. Membrane budding can also occur, wherein the plasma membrane protrudes outward, and the cytoskeleton (primarily actin) reorganizes, allowing the budding vesicle to be pinched off. This process is regulated by GTPases such as ARF6, Rab, Rac1, and RhoA [10,20]. Apoptotic bodies are the largest type of vesicles, 1000–5000 nm released by cells undergoing programmed cell death [17,[21], [22], [23]]. Unlike exosomes and microvesicles, apoptotic bodies contain DNA fragments, organelles, and proteins specific to apoptotic cells. Apoptosis begins with the apoptotic cascade, regulated by caspases that activate proteins such as Rho kinase and plexin B2, leading to membrane contractions and vesicle formation [24]. This process is tightly controlled by cytoskeletal proteins and regulated by the activation of Rho kinase and other apoptotic factors [10,25]. Apoptotic bodies can be recognized and absorbed by macrophages and other phagocytic cells involved in clearing off dying cells [26].

HMEVs contain numerous proteins, lipids, and nucleic acids, including microRNA, micro ribonucleic acid (miRNA), which can transfer intercellular signals and modulate the infants’ immune responses [[27], [28], [29], [30]]. Milk-derived EVs are crucial for gut development, especially through the presence of miRNA, which supports the maturation of the intestinal epithelium and homeostasis [31]. The functional diversity of HMEVs also includes protection against viral pathogens such as rotaviruses, HIV-1, and [32,33]. EVs can bind to dendritic cell receptors, blocking viruses from entering target cells [27,34]. The composition of HMEVs can be influenced by the mother’s health status, including stress, obesity, gestational diabetes, or premature birth, suggesting the possibility of adjusting the molecular signals transmitted to meet the developmental needs of the infant [10].

## Physiology and Health Ramifications of HMEVs

HMEVs play a crucial role in modulating the infant’s immune system. They contain various miRNAs and proteins that can influence immune cell maturation and function [35]. For instance, miRNAs in HMEVs have been shown to regulate the expression of genes involved in immune responses, thereby enhancing the infant’s ability to fight infections [35]. The consumption of HM is associated with a lower risk of various diseases, including diarrheal disease and respiratory infections [10]. HMEVs may contribute to these protective effects by transmitting bioactive molecules that enhance the infant’s immune defenses and overall health [10].

HMEVs have a significant impact on gut health, contributing to the development and maturation of the gastrointestinal tract. They provide growth factors and other signaling molecules that promote the proliferation and differentiation of intestinal cells, aiding in the overall development of the gut. HMEVs also contain bioactive molecules such as miRNAs, proteins, and lipids that can influence the composition and activity of the gut microbiota. These vesicles help establish a beneficial microbial community in the infant’s gut, which is crucial for digestion, nutrient absorption, and immune function [36].

HMEVs are also involved in the development of the nervous system. They contain miRNAs that can influence the formation of neuronal synapses and neural pathways [35]. This is critical for cognitive development and the establishment of neural networks during early life [35].

## Isolation of HMEVs

Isolation of HMEVs is an essential task in scientific research and clinical applications. Some of the described isolation techniques are successfully used to isolate EVs from other materials, and they show potential for use in the isolation of EVs from HM. The choice of the HMEV isolation method depends on the research goals, available resources, and specific requirements. Each of these methods has its advantages and limitations that need to be considered (Table 1 [34,[37], [38], [39], [40], [41], [42], [43], [44], [45], [46], [47], [48], [49], [50], [51], [52], [53]]). The isolated HMEVs can undergo further analyses.

**TABLE 1:** Comparison of human milk extracellular vesicle isolation methods.

Method of isolation	Type of uses properties	Time of the whole experiment	Advantages	Disadvantages	References
Ultracentrifugation	Density	>24 h	It is a commonly used standard method for isolating exosome vesicles.	The method is less effective when analyzing viscous fluids; for milk samples, dilution will be needed.	[38,39,40]
Density Gradient Centrifugation	Density	∼18 h	It is helpful in separating low-density EVs from other vesicles, particles, and impurities.	It is time-consuming and highly sensitive to centrifugation duration, with precise loading and unloading of the density gradient being difficult to manage.	[39,41,42]
Membrane Filtration	Size-based	∼2 h	Filtration enables the separation of small particles and soluble molecules from exosomes, and during the process, the exosome population is concentrated by the filtration membrane.	Exosomes may stick to filtration membranes, causing them to be lost for further analysis, and applying force to push liquid through membranes could deform or damage the exosomes.	[38,45,46]
Size Exclusion Chromatography	Size-based	∼8 h	This technique allows precise separation of large and small molecules, and different solutions can be applied. Unlike centrifugation, it does not damage the structure of exosomes due to shearing forces.	The method requires long processing times, limiting its suitability for handling multiple samples.	[37,49,50]
Electrophoretic filtration	Electric charge	∼2 h	This technique is relatively recent and could offer greater selectivity compared to other size-based separation methods.	Effectiveness of the method relies on the electric charge and properties of HMEVs, requiring precise adjustment of electrophoretic filtration conditions for each sample.	[47,48]
Microfluidics	Size-based, electric charge	∼2 h	The method enables high-purity EV isolation, low volume usage, high sensitivity, reduced costs, and efficient delivery of results.	The method has a low isolation capacity, lacks standardized global protocols, and requires highly specialized technical expertise.	[34,51]
Immunoisolation with micro- and nanoparticles	Chemical bounds	∼2 h	This approach allows for the isolation of either all exosomes or selective subtypes, and it is also useful for characterizing and quantifying EV proteins.	It is not suitable for large sample volumes, and isolated vesicles may lose their functional activity.	[43,52,53]
Polymer precipitation	Physical	∼2 h	The main benefit of the precipitation method is that it has a mild impact on the isolated exosomes.	This method can co-isolate nonvesicular contaminants like lipoproteins, and the presence of polymeric substances might interfere with downstream analyses.	[37,44]

Abbreviations: EV, extracellular vesicle; HMEV, human milk extracellular vesicle.

### Ethical considerations

The studies involving the isolation of EVs from HM are conducted following strict ethical guidelines to ensure the protection of donors and the integrity of the research. Ethical approval must be obtained from the relevant institutional review boards or ethics committees before the commencement of the studies. Milk donors are provided with comprehensive information about the study, including its purpose, procedures, potential risks, and benefits. Written informed consent is obtained from all participants, ensuring they fully understood and voluntarily agreed to participate. The studies are designed to minimize any potential risks to the donors. The collection of HM is performed in a noninvasive manner, and all procedures adhere to the highest standards of safety and hygiene [37].

Maintaining donor privacy and confidentiality is a critical aspect of the studies. Several measures were implemented to protect donor information. Donor samples are anonymized by assigning unique identification codes, ensuring that personal identifiers are not linked to the samples or data. All data, including consent forms and research findings, are stored securely in password-protected databases. Access to this data are restricted to authorized personnel only. Researchers involved in the studies may be asked to sign confidentiality agreements, committing to protect the privacy of the donors and the confidentiality of the data [37].

### Preliminary purification of EVs fraction

Human breast milk is a complex biological fluid containing various cells, proteins, and lipids that can interfere with the isolation and characterization of EVs. To improve the HMEVs isolation process, it is advisable to remove the problematic components.

**FIGURE 1:**
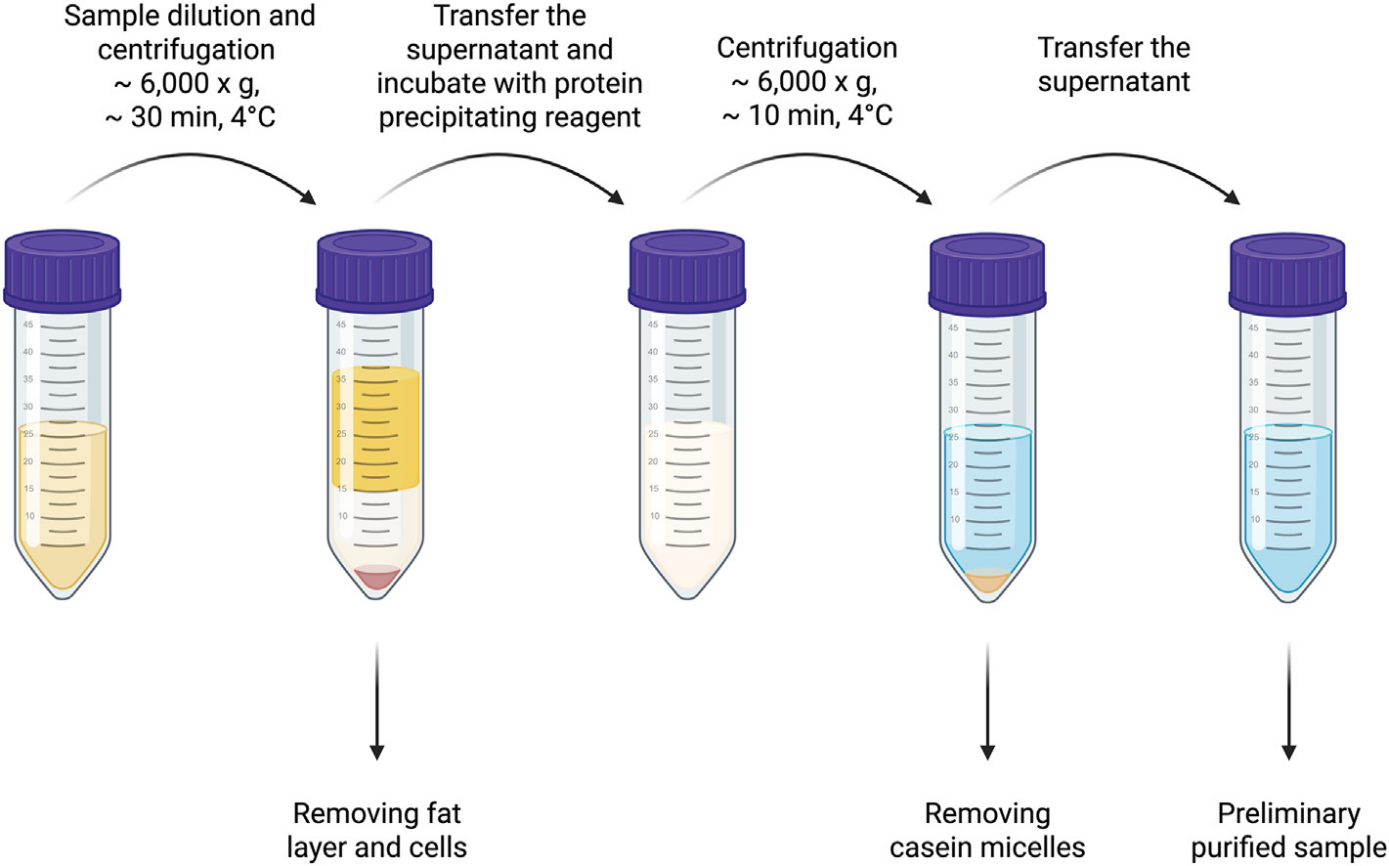
Human milk preliminary purification procedure scheme [[40], [43], [44]]. Created with Biorender.com.

In the first step of preliminary purification (Figure 1), HM undergoes low-speed centrifugation (400 x *g*) for a short duration (∼30 min). The purpose of this step is to remove the largest milk constituents, such as microbial and somatic cells, which settle in the form of pellets at the bottom of the centrifuge tube.

In the next step, the centrifugation speed is increased to 2000 x *g*. This stage aims to separate larger particles, vesicles, and membrane fragments from smaller HMEVs. Larger structures again settle at the bottom of the centrifuge tube, whereas the remaining supernatant contains smaller HMEVs like microvesicles and exosomes. At this stage, it is also possible to remove the milk fat layer settled on the top of the tube mechanically or by using filters. As the size ranges of milk fat globules and HMEV particles overlap, fractions of isolated HMEV particles from milk may be contaminated with milk fat globules [54]. To avoid this, the milk can undergo further centrifugation at a higher speed, for example, 3000 x *g* [38].

The last step of preliminary purification is aimed at removing casein, a milk protein that forms spherical colloidal aggregates known as casein micelles (diameter from 20 nm to 600 nm) [55], which also overlap in size with EVs. Centrifugation at high speed in a range of (20,000–75,000 x *g*) removes most of the casein micelle fraction; however, it is crucial to note that the removal of this layer may affect the quantity of EVs because of similar size to HMEVs. To improve the elimination of milk proteins, EDTA or sodium citrate can be used in the presence of calcium ions chelate casein micelles [56,57]. The samples are incubated with 20 mM EDTA or 10 mM sodium citrate solution for 10 min at 4°C [56] or overnight at 25°C [57], and then they are centrifuged (12,000 x *g*; 20 min; 4°C). Chymosin can also be used because it hydrolyzes and breaks down casein. The samples are incubated with 0.05% chymosin in 0.3% CaCl2 for 30 min at 4°C and then centrifuged (16,500 x *g*; 30 min; 4°C) [58]. Overnight incubation with sodium phosphate (20 mM) and the subsequent centrifugation (5000 x *g*; 30 min; 4°C) may also be used to induce casein micelles precipitation [57]. Published data showed that acidification may impact the morphology of HMEVs, whereas EDTA and chymosin caused slight changes in EV number [56].

By implementing these preliminary purification steps, we can significantly enhance the purity of the isolated EVs, thereby improving the reliability and reproducibility of subsequent characterization and functional studies.

### HMEVs isolation methods

There are several described methods of EV isolation, and they can be basically divided into 3 groups: density-based, size-based, and affinity-based isolation methods.

#### Density-based isolation

##### Differential ultracentrifugation

**FIGURE 2:**
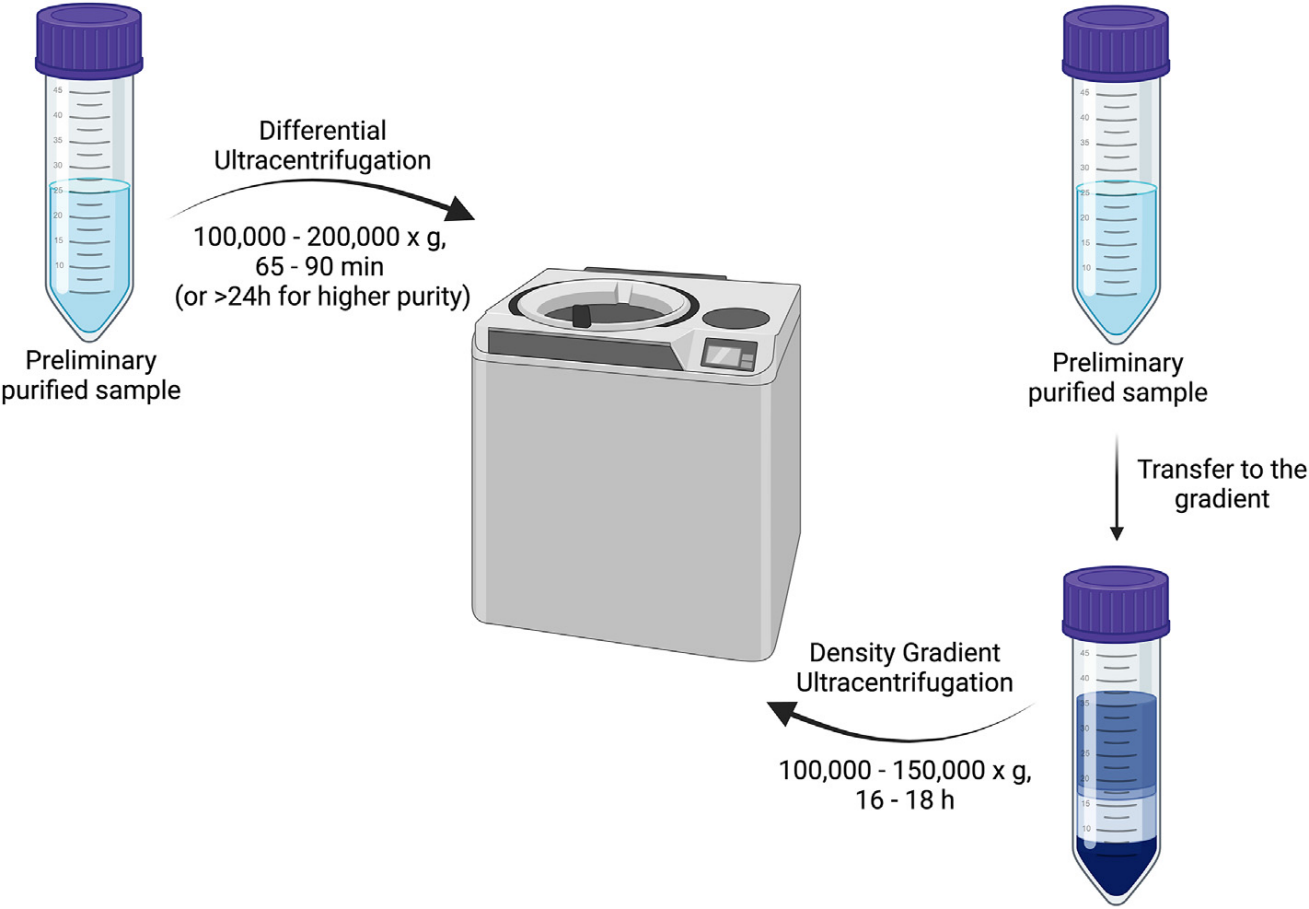
Ultracentrifugation methods for EVs isolation [[47], [49], [51], [55]]. Created with Biorender.com.

The ultracentrifugation method is a traditional and widely used technique for HMEV isolation (Figure 2), often referred to as the “golden standard” for EV isolation. It separates HMEVs based on their density by centrifuging the sample at ultrahigh speeds. The ultracentrifugation speed typically reaches values between 100,000 and 200,000 x *g*, and the time duration ranges from 65 to 90 min [5,[38], [39], [40],^59^]. To obtain a fraction of pure EVs, it is necessary to centrifuge repeatedly at increasing speed, which can take ≤24 h in total. In this step, exosomes and sediment at the bottom of the centrifuge tube, due to their higher density compared to other milk components, are ∼1.11–1.19 g/mL [60].

It is important to note that ultracentrifugation is a complex and time-consuming process that requires specialized equipment and expertise. Therefore, scientists are also developing more modern and efficient methods for isolating HMEVs that can be applied in clinical settings and laboratory research. When using this method, it is common to use additional membrane filters (usually of 0.20–0.22 *μ*m) to make sure that the final fraction of exosomes is obtained specifically [5,38,39].

##### Density gradient ultracentrifugation

This technique involves ultracentrifugation of a sample containing HMEVs in a density gradient (Figure 2) [39,41,61]. HMEVs move up the gradient based on their density, enabling their separation from other components, such as proteins.

To prepare the density gradient, solutions of sucrose (2–0,4 M) [42] or iodixanol (40% (wt/vol) or 60% (wt/vol) solution of iodixanol in water) [41,62] are typically used. The density gradient is layered in tubes or inserts, and it must be precisely controlled and adjusted according to the requirements. The sample is applied to the top of the density gradient. The tube or insert with the loaded sample is placed in an ultracentrifuge and subjected to high-speed centrifugation (usually from 100,000 x *g* to 150,000 x *g* or more) for a specified time duration (e.g., 16–18 h) [39,41,43]. During ultracentrifugation, HMEVs migrate down the density gradient depending on their density until they reach their respective positions in the gradient. After ultracentrifugation, the sample is fractionated [40,61,63]. Each of these fractions contains HMEVs with similar density. This is particularly important in scientific research, where various types of HMEVs may have different compositions or functions.

The density gradient ultracentrifugation is valuable as it allows for the precise separation of different fractions of HMEVs based on their density. It’s worth noting that this technique requires specialized equipment (ultracentrifuge), and the control of parameters such as gradient density and centrifugation speed is crucial for its effectiveness.

#### Size-based isolation

##### Polymer precipitation. (e.g., polyethylene glycol)

**FIGURE 3:**
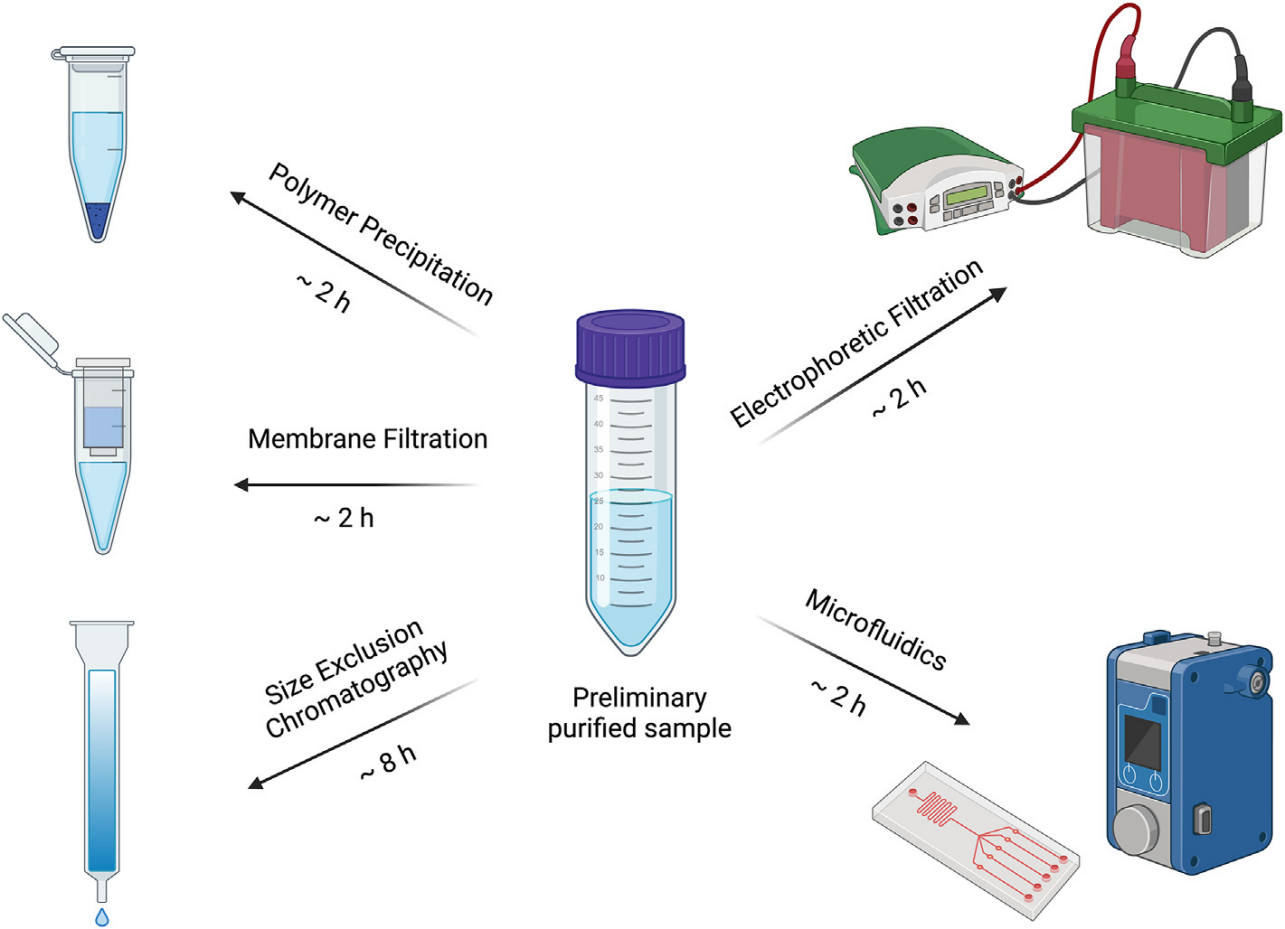
Size-based isolation methods for HMEVs [[39], [41], [58], [64], [67], [79]]. Created with Biorender.com.

Polymer precipitation, using polyethylene glycol (PEG), is 1 of the methods for the isolation of HMEVs (Figure 3), which is gaining widespread interest from milk or other biological fluids. In this method, PEG acts as an osmotic agent counteracting the solubility of particles, leading to the aggregation and precipitation of HMEVs from the solution. PEG is available in various concentrations that can be chosen for a specific application. After adding PEG, the sample is incubated for a certain time, typically several hours or overnight. During the incubation, PEG binds to HMEVs, causing their precipitation. After, the sample is centrifuged, usually at low speeds (4000–18,000 x *g*) (unpublished data). This allows the separation of the precipitated material from the remaining solution. The pellet containing the precipitated EVs is collected and re-suspended in an appropriate buffer or medium [37,44].

Polymer precipitation has several advantages, including simplicity and the fact that ultracentrifugation is not necessary. It is a relatively fast method of HMEV isolation, making it attractive for clinical research and diagnostics. PEG is commonly used in commercially available kits (e.g., ExoQuick) designed for EV isolation. The method was applied for the isolation of EVs from HM [37].

However, as with all other methods, this one also has some limitations. Firstly, the nonspecific nature of PEG-induced precipitation results in the co-isolation of nonvesicular contaminants, especially soluble proteins and protein aggregates [64]. Another important limitation concerns the impact of residual PEG on downstream applications. Although the protocol includes optional wash steps (e.g., ultracentrifugation or secondary precipitation with lower PEG concentration), incomplete removal of PEG can interfere with sensitive analytical techniques such as mass spectrometry. To mitigate this, additional cleanup procedures, such as gel purification or chromatography, may be required, introducing complexity and extending processing time [64].

##### Membrane filtration

Membrane filtration is an effective method that allows the separation of EVs from other sample components based on particle size (Figure 3). The selection of an appropriate filtration membrane with the right pore size is crucial. Membrane filters are available in various pore sizes ranging from 0.2 to 0.8 μm [38,65]. Particles larger than the membrane pores are retained and will not pass through to the other side, whereas HMEVs will be allowed to pass. Membrane filtration is an efficient and relatively simple method for isolating HMEVs, allowing the preservation of their integrity and structure [38,45,46]. This is particularly useful when rapid isolation of HMEVs is required for research or diagnostic purposes. It should be noted that this method of isolation may lead to the loss of a significant fraction of HMEVs, which will be larger than the filter pore size. The choice of an appropriate filtration membrane depends on the expected size of HMEVs and the quantity of the milk sample to be filtered [66]. A sequential application of filters with varying pore sizes can effectively purify the HMEVs fraction; however, this approach often leads to a substantial reduction in particle count. It is also important to monitor the quality of isolation and perform appropriate analyses to confirm the presence and purity of the isolated HMEVs.

##### Electrophoretic filtration

The electrophoretic filtration method is an advanced technique for isolating HMEVs (Figure 3) from biological fluids that use an electric field to move and concentrate HMEVs on special membranes based on their electric charge and size. This method is relatively new and may be more selective than other size-based separation methods [47].

A sample of HM is applied to a chamber with a special membrane called an electrophoretic filtration membrane. Cho et al. [48] (2016) used a membrane composed of polycarbonate track-etched material with a pore size of 30 nm. This material offered both structural stability and selective permeability, ensuring the effective retention and concentration of nanoscale EVs during the isolation process. Next, the electrophoretic separation was conducted using a TAE (*tris*-acetate-EDTA) buffer, providing suitable ionic strength and pH stability for consistent EV migration. To maintain isotonic conditions and enhance biological compatibility, a sugar-based buffer composed of 4-(2-hydroxyethyl)-1-piperazineethanesulfonic acid (HEPES), sucrose, and D-glucose was incorporated into the system. This formulation helped to preserve vesicle morphology and functionality throughout the isolation process. An electric potential of 100 V was identified as optimal, allowing for effective electrophoretic migration of EVs without inducing thermal degradation. The resulting power output was maintained at 0.45 W, which represented the upper threshold to prevent overheating during operation. After the selective separation of HMEVs on the membrane, elution can be performed. Elution involves washing HMEVs off the membrane using appropriate solutions or buffers. For the elution of EVs from the membrane following separation, phosphate-buffered saline was employed. Phosphate-buffered saline enabled efficient recovery of intact EVs from the membrane surface, facilitating their downstream analysis or application [48]. However, this method is more advanced and requires specialized equipment and expertise in electrophoretic filtration. It’s important to note that the effectiveness of this method depends on the electric charge and other properties of HMEVs, so precise adjustment of electrophoretic filtration conditions to the specific sample is necessary. This method was, to date, only applied to the isolation of EVs from human blood samples, but it has the potential to be used for HM [48].

##### Size exclusion chromatography

Size exclusion chromatography uses columns filled with a porous gel polymer, where small particles such as HMEVs are separated from larger particles (Figure 3). The column filling has pore sizes tailored based on the fraction 1 intends to retain. For the separation of EVs, columns with pores in a range of 35–220 nm are employed [49,50]. The sample is then eluted from the column. The obtained fraction contains pure HMEVs.

This is 1 of the methods for isolating HMEVs, which is relatively user-friendly and does not require any special equipment like an ultracentrifuge. It allows the selective isolation of HMEVs based on their size, which can be useful in studies focusing on specific HMEV subpopulations [49]. However, this technique may not be as precise as ultracentrifugation since the separation relies on differences in size, which may lead to some contamination with other particles. This method has been applied for the isolation and purification of EVs derived from bovine and HM [37].

##### Microfluidics

Microfluidics utilizes microscale systems and microchannels placed on the microchip made of glass or polymer that allow the control of sample flow (Figure 3). The milk sample is introduced into microchannels where the flow, separation, and sorting of HMEVs can be manipulated [51]. Among the microfluidic strategies for EV isolation, 2 major categories can be identified: those based on surface antigen recognition and those utilizing physical characteristics such as size or density.

The first group includes microfluidics-based immunoaffinity capture systems, which employ antibodies targeting specific membrane antigens expressed on EVs. These antibodies may be immobilized either on the inner surface of microfluidic channels or on carrier beads, such as magnetic nanoparticles. For instance, Kanwar et al. [67] (2014) developed the ExoChip platform that utilizes anti-CD63 antibodies for EV capture, whereas Chen et al. [68] (2010) introduced a herringbone-grooved microchannel design that enhances capture efficiency by inducing microscale turbulence. Although microfluidics-based immunoaffinity capture methods offer high specificity and facilitate downstream analysis, they are inherently limited to subpopulations of EVs bearing known surface markers [69,70].

The second group comprises microfluidic fractionation approaches that exploit EVs’ biophysical properties. Filtration-based systems such as Exodisc [71] or the dual-filtration chip described by Liang et al. [72] (2017) employ nanoporous membranes with defined pore sizes (e.g., 600 nm and 20–30 nm) to selectively retain vesicles within a target size range. Other approaches include nanowire traps [73], deterministic lateral displacement using pillar arrays [74], and viscoelastic flow-based separation, which enables label-free isolation of EVs with high purity (>90%) [75].

Acoustofluidic technologies represent another innovative solution, using acoustic forces such as surface acoustic waves to manipulate and separate EVs based on their size and mechanical properties [71,76]. These systems are contact-free, highly biocompatible, and readily integrable with downstream analytical modules. Isolation of HMEVs using microfluidics remains an area of research, and various techniques and devices are continuously being developed to enszable more advanced isolation methods [34].

#### Affinity-based isolation

**FIGURE 4:**
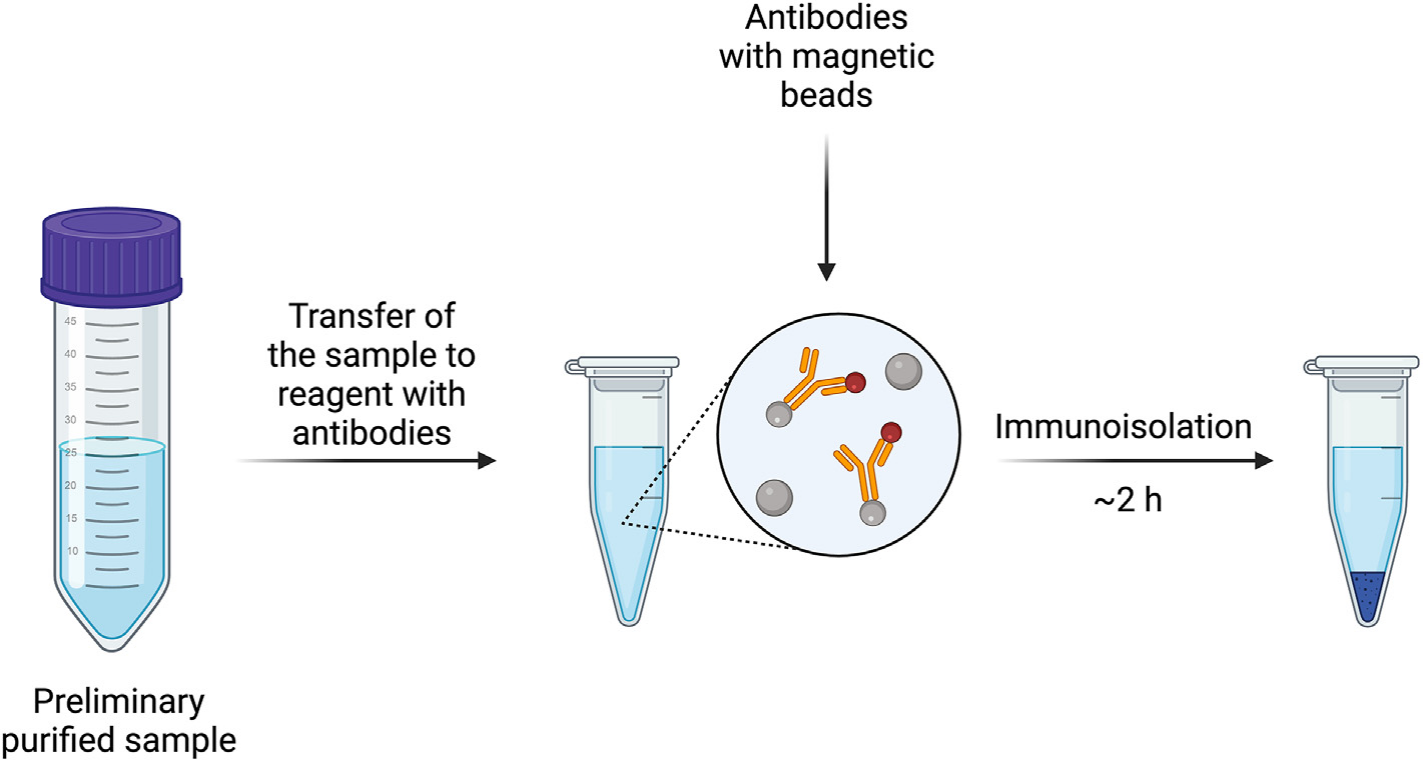
Immunoisolation [[55], [79], [83]]. Created with Biorender.com.

Immunoisolation is an advanced technique that utilizes antibodies specific to certain surface proteins of HMEVs to selectively isolate these vesicles (Figure 4). They may be attached to carriers, such as magnetic beads or other microparticles, facilitating the isolation of HMEVs [2,77]. Prepared magnetic beads with antibodies are added to the HM sample containing HMEVs. After a short incubation time, magnetic beads or other carriers are separated from the rest of the sample using a magnet or magnetic platform [77]. Several washing cycles are then performed to remove nonspecifically adhering components. Next, HMEVs are eluted from the carriers by breaking the antigen-antibody bond.

The selection of appropriate antibodies is crucial because the specificity of the antibodies ensures that the isolation targets the desired subpopulations of HMEVs with precision. Isolation of HMEVs from milk using antibodies may involve the use of antibodies directed against proteins characteristic for EVs present in HM, such as monoclonal antibodies: anti-CD81, anti-CD63, anti-ICAM-1, anti-Lactadherin, and anti-MUC [5,78,79].

The immunoisolation method of HMEVs has some limitations, such as the requirement for the availability of specific antibodies and the risk that isolated HMEVs may be damaged during the process. The need for appropriate ligands and micro- and nanomagnetic particles may significantly increase the analysis costs. It may also be more challenging to perform with samples containing high lipid content, which can affect the ability of magnetic particles to bind to HMEVs without proper preliminary purification. Another limitation of using immunoisolation for HMEVs is that it necessitates thorough validation to ensure specificity and minimize cross-reactivity with other milk components. Antibody validation is crucial to confirm that antibodies specifically bind to their target antigens without cross-reacting with unrelated proteins. It can be done by using immunoprecipitation followed by mass spectrometry. This technique helps to identify the proteins that are bound by the antibody, ensuring that the antibody is specific to the target protein. Cross-reactivity occurs when antibodies bind to nontarget proteins that share similar epitopes. Studies have shown that antibodies can cross-react with proteins in complex biological fluids like milk [80]. For instance, IgG antibodies against milk proteins have been observed to cross-react with other antigens, which could potentially interfere with the isolation of EVs [80]. To address these concerns, it is essential to perform cross-reactivity tests (immunoblotting or ELISA) to test the antibodies against a panel of milk proteins and identify any nonspecific binding. Additionally, the use of multiple antibodies against different markers (e.g., CD9, CD63, CD81) can increase the specificity of EV isolation and optimize protocols to minimize non-EV components that could interfere with antibody binding [81]. By implementing rigorous validation methods and addressing potential cross-reactivity, the specificity and reliability of antibodies used in EV isolation from HM can be ensured. But even with those disadvantages, this method is highly specific, rapid, and efficient.

#### Isolation using commercial kits

There are some commercial kits for the isolation of EVs from HM and other biological fluids available on the market, making the isolation process easier and faster compared to other described methods. These kits are based on different principles. Some of them utilize centrifugation techniques, whereas others rely on adsorption or other mechanisms like immunobinding. When selecting a commercial kit for isolating HMEVs, it is important to select it based on the specific research purposes. It’s also crucial to follow the manufacturer’s protocols to ensure effective isolation and purification of HMEVs.

The availability of commercial kits for isolating HMEVs may change over time, and the choice depends on specific companies and the market. Table 2 presents some examples of HMEV isolation kits that are now available on the market.

**TABLE 2:** Commercial kits for human milk extracellular vesicles isolation.

Company	Trade name	Utilized technology	Types of EVs	Yield	Purity	Ease of use
Qiagen	ExoRNeasy kit	Membrane filtration	Exosomes	High	High (excludes nonvesicular RNA)	Easy, convenient on-column procedure
	ExoEasy kit			High	High (better than ultracentrifugation)	Easy, fast, and consistent spin-column procedure
Norgen Biotek	Exosome purification kit	Labeled ligands	Exosomes	High	High (removes RNA-binding proteins)	Easy, simple spin-column chromatography
	Exosome RNA purification			High	High (high integrity RNA)	Easy, all-in-1 system, no special instrumentation needed
System Biosciences	ExoQuick	Precipitation	All	High	High (reduces the content of albumins and Igs	Easy, fast processing time
	Exo-Flow	Density differences		High	High (low background binding)	Easy
	ExoELISA	Immunoassay	Specific	High	High	Easy, fast assay
Thermo Fisher Scientific	PureExo exosome isolation kit	Ligands	Exosomes	High	High	Easy, no ultracentrifugation required
	PureExo total RNA isolation kit	Extraction	Exosomes	High	High-quality RNA	Simple and rapid protocol
Bio-Techne	Exo-spin kits	Ultracentrifugation with chromatographic columns	All	High	High (low concetrations of protein and rRNA contamination)	Simple and reliable size exclusion chromatography

Abbreviations: ELISA, enzyme-linked immunosorbent assay; EV, extracellular vesicle; Ig, immunoglobulin; RNA, ribonucleic acid; rRNA, ribosomal RNA.

## Characterization Methods

The next step after the isolation of HMEVs is their characterization, which may include their morphological analysis and quantitative and qualitative analysis (Table 3 [43,44,46,57,[82], [83], [84], [85], [86], [87], [88], [89], [90], [91], [92], [93], [94], [95], [96], [97], [98], [99], [100]]).

**TABLE 3:** Comparison of human milk extracellular vesicles characterization methods.

Method of characterization	Source of data	EVs characteristic	Advantages	Disadvantages	Sensitivity	Specificity	Biogenesis confirmation	References
Flow cytometry	Light scattering, fluorescence	Size, shape, quantity	Rapid measurement and processing of HMEVs in fluid allows multiparametric analysis.	Limited resolution for small EVs: requires optimization to reduce background noise.	High	High (when using immunologic markers)	Yes (when using immunologic markers)	[80,83,84]
Dynamic light scattering	Light scattering	Size distribution	Simple and noninvasive method for measuring particle size ranging from 1 nm to 6 *μ*m.	Designed for homogeneous particles: challenges arise when larger particles are present in the sample; cannot differentiate between EVs and other particles of similar size.	High	Limited	No	[57,88]
Nanoparticle tracking analysis	Light scattering	Size, concentration	It is effective in analyzing size distribution and concentration in polydisperse samples in real-time analysis.	In nonuniform systems, light scattering from protein aggregates or other particles may interfere with the accurate measurement of EV concentration and size distribution.	High	Limited	Yes	[57,91,93]
Electron microscopy (SEM/TEM)	Electron beam	Size, shape	SEM allows for 3D imaging of the external features and surface topography of EVs. TEM provides 2D images, revealing the internal structure of EVs. Cryo-TEM offers high-resolution imaging without damaging the biological structure by flash-freezing samples, maintaining the integrity of EVs.	Requires extensive sample preparation (fixation, which can alter their natural state) and is labor-intensive and time-consuming.	High	High	No	[44,80,91]
Atomic force microscopy	Atomic beam	Size, shape, structure	Allows accurate measurement of sample dimensions at the atomic level and can measure isolated samples in their natural state with little to no preparation.	Slow scanning speed: immobilization and ligand binding might be necessary to prevent deformation.	High	High (when using fluorescent labeling)	Yes	[80,98,101]
Confocal microscopy	Light scattering	Count, size, structure, distribution	With lipophilic dyes, EVs can be labeled easily without antibodies, simplifying sample preparation and maintaining the integrity of vesicle membranes.	Confocal microscopy’s resolution is diffraction-limited (around 200–250 nm laterally), which may be insufficient for accurately imaging individual EVs smaller than 150 nm, potentially resulting in a lack of structural detail.	Limited	High (when using fluorescent labeling)	Yes (only using immunologic markers)	[103,104]
Tunable resistive pulse sensing	Electric resistance	Size, quantity	Adjustable pore sizes allow for accurate measurement of various particle sizes, including small EVs (as small as 50 nm).	Pores may become blocked by particles, disrupting the measurement process.	High	High	No	[95,96]
Western blotting	Chemiluminescence, colorimetric	Identification of biomarkers	The method enables the detection of specific proteins using antibodies, allowing for precise identification of target proteins like HMEV markers (CD9, CD63, and CD81).	The technique is multi-step and can be labor-intensive, requiring time for sample preparation, electrophoresis, transfer, and detection.	High	High (dependent on antibody specificity)	Yes	[46,105,106]
ELISA	Colorimetric, fluorescence	Quantity	ELISA provides precise detection and quantification of specific HMEV subgroups, like exosomes, by targeting proteins such as tetraspanins.	The method depends on antibodies that specifically bind to HMEV, which may limit its versatility for different HMEV subgroups and potential for antibody cross-reactivity.	High	High	Yes	[43,108,109,110]
Lateral flow immunoassay systems (LFIA)	Visual	Size, concentration	The test results can be easily read visually or with a reader, facilitating straightforward interpretation.	LFIA has not been applied to identify EVs from mammalian milk, indicating limited applicability in some contexts.	Moderate	Moderate	Yes	[112,113]

Abbreviations: ELISA, enzyme-linked immunosorbent assay; EV, extracellular vesicle; HMEV, human milk extracellular vesicles.

### Flow cytometry

In flow cytometry (FC), the tested sample passes through a narrow stream of fluid where HMEVs move individually under the laser. Various parameters of HMEVs, such as size, shape, and morphological complexity, may be analyzed using FC. Modern flow cytometers normally can detect particles larger than 500 nm, and only some with enhanced parameters can identify particles with a diameter of 200 nm; thus, for the detection of exosomes, which are smaller than 200 nm, the enhanced flow cytometer is necessary [82,101].

There is an alternative FC method that uses detection based on a fluorescence parameter rather than just light scattering to increase the separation of HMEV signals from the background [39,83,102]. For this analysis, HMEVs are stained using fluorochromes (MEM-Glow) or immunologic markers (like CD63) specific to a particular antigen or structure [84].

FC can also be used for quantitative analysis of HMEVs by adding a known number of fluorescent latex beads as an internal standard or using tubes containing a predetermined number of beads [103].

### Dynamic light scattering

Dynamic Light Scattering (DLS), also known as photon correlation spectroscopy, is 1 of the most popular methods for characterizing EVs. DLS analyzes the thermal motion of particles in a solution induced by the Brownian motion of these particles. During the measurement, a laser emitting light is directed onto a sample containing HMEVs, and based on the fluctuations in light scattering, the average particle size and the size distribution of particles in the sample can be calculated [45,82,104].

It is a straightforward method for measuring particle size without visualizing them, so it does not provide any biochemical information regarding the biogenesis of EVs [105]. The advantage of this method is its ability to measure particle sizes ranging from 1 nm to 6 *μ*m; however, it is designed for measuring the size of homogeneous particles in suspension [85]. In cases where large particles are present in the sample, recognizing and estimating the size of smaller particles becomes more challenging, and there is a risk that HMEV populations need to be preseparated using other techniques [106,107].

### Nanoparticle tracking analysis

In nanoparticle tracking analysis (NTA), the impact of particle size on its diffusion rate in a static solution is utilized, and this change in rate is induced by Brownian motion. This relationship enables the estimation of the diffusion coefficient and the size of individual observed particles by analyzing the trajectory of their movement [86]. The process involves recording time sequences of particles undergoing Brownian motion using light scattering NTA imaging or fluorescence emission (Fl-NTA) on a specially prepared recorder (detector) or a photosensitive camera [18]. By analyzing a significant number of individual trajectories, it is possible to estimate particle concentration and size distribution, even in polydisperse samples [86].

NTA analysis is a technique limited by the continuous diffusion of vesicles entering and exiting the detection focal region, leading to the capture of relatively short particle trajectories [86]. Due to this phenomenon, generally high statistical uncertainties occur, which can be reduced by analyzing significantly longer particle trajectories [108]. It should also be considered that scattered light may interfere with the assessment of the concentration and size distribution of HMEVs in nonuniform systems due to the presence of other scattering sources, such as protein aggregates [87]. Therefore, special attention should be paid to the purity of the analyzed HMEVs.

Despite its limitations, NTA allows for a rapid assessment of the size, distribution, and concentration of HMEVs, making it widely used in HMEVs research [39,45,104]. The Fl-NTA technique is used to differentiate EVs from other particles by specifically tracking fluorescently labeled HMEVs [18,109]. Due to the necessity of using very bright and photostable fluorescent markers (e.g., quantum dots) for detection and avoiding excessive bleaching during measurements [109], Fl-NTA, unlike the scattering NTA technique, has not become 1 of the standard methods for EV determination.

### Tunable resistive pulse sensing

Tunable resistive pulse sensing (TRPS) is a technique that enables precise analysis of the size and number of particles in a solution. In this method, a sample is introduced into a measurement chamber and pumped through a microporous membrane-containing pores of controlled size while an electric current flows through the membrane. As particles pass through the micropores, they temporarily alter the electrical resistance of the current flow. These changes are recorded and analyzed. Based on the duration and amplitude of the resistance changes, TRPS allows for the determination of the size and quantity of particles in the sample. Because the pores on the membrane are tunable, the technique can be adjusted to accurately measure various particle sizes, including small EVs with a diameter of ∼50 nm [85,88].

TRPS enables the characterization of each particle in the sample, including colloidal particles, various nanoparticles, and biomolecules in suspension, distinguishing this technique from others. There are also some limitations associated with this technique, such as the potential for pore blockage by particles and situations where the target particles are too small to be detected against the background noise of the measurement system [85]. Addressing these issues can be achieved through optimizing system parameters, such as noise reduction, establishing sensitivity limits, improving measurement accuracy, and selecting appropriate pore sizes [88].

TRPS has been utilized in the characterization of EVs from cow milk, but it has not yet been employed for the identification of HMEVs [89,110].

### Scanning electron microscopy and transmission electron microscopy

The most used and direct method for determining the size and morphology of individual EVs is electron microscopy (EM). In this technique, an electron beam is used instead of light, allowing for high-resolution imaging of objects on the nanoscale [82]. Two frequently employed types of EM are SEM and TEM.

SEM provides images of the surface topography of HMEVs by scanning the surface with a focused electron beam and detecting the secondary electrons emitted by atoms in the analyzed area. This technique produces 3D images. SEM allows for the visualization of the external features of HMEVs [39,104].

TEM utilizes electrons that pass through the sample to create a 2D image of HMEVs. The images obtained by TEM are based on the transparency of features in the examined object to the electron beam, providing information about the internal structure [86]. The thickness of the analyzed material is limited by the ability of electrons to effectively pass through objects with a thickness of 50–500 nm, depending on the electron beam power.

One limitation of EM for biological objects like cells and also HMEVs is the requirement for imaging in a vacuum, often necessitating sample fixation and dehydration, which can cause vesicle damage [86]. Cryogenic EM techniques, such as cryo-TEM, have been developed to address this limitation. Cryo-TEM involves imaging an ultra-thin, flash-frozen layer of a liquid suspension of HMEVs at very low temperatures (<−100°C) [40]. This technique allows for high-resolution imaging of biological structures without damaging their integrity and is commonly used for determining the ultrastructure of HMEVs. Additional immunogold labeling in cryo-TEM enables the identification of specific HMEV subtypes in a mixture. Cryo-TEM is considered 1 of the most accurate methods for HMEV size characterization. However, it does not provide information about HMEV concentration due to the potential effects of HMEV interactions with the TEM grid and sample blotting [82,86].

### Atomic force microscopy

Atomic Force Microscopy (AFM) is a technique that detects and records interactions between a measurement tip and the sample surface. This method enables imaging of the morphology of particles like HMEVs, including their shape, structure, and nanoscale details, as well as precise size measurements.

In AFM, a probe, typically a sharp tip such as a metal needle or thin fiber, is placed at the end of a lever. The lever supports the probe and enables its movement in 3D. AFM measures forces between the probe and the sample at the atomic level. As the probe approaches the sample surface, intermolecular forces between atoms on the probe and atoms on the sample influence the probe’s movement. When forces between the probe and the sample change, the lever undergoes deformation. This deformation is measured by a detector that records the lever’s displacement. During scanning, the distance between the probe and the sample is adjusted to maintain a constant atomic-level force, allowing for high-resolution surface imaging. Data collected during scanning are used to create a 3D image of the sample surface. Analysis of this data provides information about the sample’s topography, roughness, and other surface properties [82,90].

An important feature of AFM is its ability to measure samples in their natural conditions, compared to SEM and TEM, with minimal sample preparation [91,111,112]. AFM enables obtaining a real 3D image of surface topography with very high resolution when HMEVs are immobilized (e.g., on a mica surface) [82]. Immobilized HMEVs may undergo deformation, which can be prevented by binding ligands to the mica surface and then binding HMEVs to these ligands, which may be specific to a particular HMEVs subtype [82,112,113].

### Confocal microscopy

Confocal microscopy enables high-resolution imaging of HMEVs by utilizing fluorescent dyes that integrate directly into vesicle lipid membranes. Lipophilic dyes bind to the lipid bilayers of HMEVs, providing clear imaging without the need for antibody labeling [92].

Using confocal microscopy with lasers at appropriate wavelengths (red and far-red) activates these dyes, minimizing tissue autofluorescence and enhancing image quality. Emission filters allow for simultaneous visualization of multiple dyes (green and blue) [92,93], facilitating HMEV analysis within the context of surrounding cellular structures. Image analysis involves quantifying HMEV count, size, and distribution, as well as colocalization studies that reveal potential interactions between HMEVs and other cellular components [92]. A key limitation of confocal microscopy is its resolution, which may be insufficient for detailed imaging of individual HMEVs smaller than 150 nm [92,93].

### Western blotting

Western blotting (WB) is a technique used to identify and analyze specific proteins in biological samples [40,94]. In the context of characterizing HMEVs, WB is commonly employed to confirm the presence of marker proteins specific to these structures, such as membrane proteins CD9, CD63, and CD81.

Following isolation, the HMEVs undergo lysis, which releases proteins that can then be prepared for electrophoresis. Next, these HMEV proteins are separated on a polyacrylamide gel based on their molecular weight, allowing for the subsequent detection of specific proteins. After separation, the proteins are transferred onto a nitrocellulose or polyvinylidene fluoride (PVDF) membrane, which facilitates their detection by antibodies. On this membrane, primary antibodies directed against HMEV marker proteins are applied, followed by secondary antibodies linked to an enzyme that allows for detection through chemiluminescent or colorimetric methods [94,95].

WB enables not only the confirmation of HMEV presence through the detection of specific markers but also the assessment of sample purity [40,94,95,114].

### ELISA

ELISA is a laboratory technique used for the detection and quantitative measurement of specific substances, such as proteins, antigens, or antibodies, in biological samples. In the context of HMEV characterization, ELISA can be employed to identify and quantify the measurement of proteins specific to these structures.

In ELISA, plates are coated with a substance that specifically binds to HMEVs, often antibodies directed against proteins present on the surface of HMEVs. Next, secondary antibodies, coupled with an enzyme, are added to the plate. These antibodies react with HMEVs bound to the plate. After washing the plate, a substrate for the enzyme is added, generating an optical signal, such as color or fluorescence, proportional to the number of secondary antibodies bound to HMEVs. The signal intensity is measured using a spectrophotometer or other appropriate measuring equipment. The quantitative measurement of the signal allows for the assessment of the amount of HMEVs present in the sample [96,97].

ELISA is frequently used due to its high specificity in detecting and quantifying specific HMEV subgroups, such as exosomes, by targeting tetraspanins [58,97,98].

#### Lateral flow immunoassay systems

Lateral flow immunoassay systems (LFIA) is a test used for the rapid assessment of the presence and/or quantity of specific biomarkers in a sample [115]. In the context of EV characterization, LFIA can be employed to identify proteins or other molecules present on the surface or within the cell membrane.

EVs can be labeled with specific markers, such as antibodies or other molecular probes. The LFIA test is designed in the form of a strip or a test card. Reagents, such as antibodies reacting with labeled EVs, are placed on this card. Labeled EVs are introduced onto the LFIA test, which consists of control and test areas. In the presence of target proteins, immunologic reactions lead to the formation of lines in the test area. After a certain incubation time, the test results can be read visually or using a reader [99,100].

The overall performance of the LFIA test is largely determined by the physical structure and chemical composition of the membrane-containing antibodies. The speed at which the sample-analyte complex is transported through the membrane determines the reaction time and, thus, the sensitivity of the test [116]. Although LFIA is a rapid diagnostic test with potential applications in identifying specific EV populations, it has not yet been utilized in the identification of EVs from mammalian milk.

## Conclusions

HMEVs are of significant interest due to their diverse origins and multifaceted roles. Predominantly produced and secreted by mammary gland epithelial cells during lactation, HMEVs also derive from other cells present in HM, such as lymphocytes, macrophages, and stem cells. Additionally, EVs from other organ cells can enter the milk *via* systemic circulation, further enriching the EV pool. HMEVs play crucial roles in infant gut development, protection against viral pathogens, and potential modulation based on the mother's health status, making them a vital component of neonatal development and health.

Research on HMEVs faces several challenges. One major challenge is the lack of standardized protocols for the isolation and characterization of HMEVs, which can lead to variability in results and hinder comparability between studies. The complexity and heterogeneity of HMEVs also pose difficulties in accurately identifying and categorizing the different subpopulations. Additionally, the small size and low abundance of certain EV subtypes require highly sensitive and specific analytical techniques, which may not be readily available in all research settings. Understanding the precise biological functions of HMEVs and their molecular cargo in the context of infant development and health is another significant challenge, necessitating advanced experimental models and long-term studies. Finally, ethical considerations related to the collection and use of HM samples must be carefully managed to ensure the protection of donor privacy and the ethical use of biological materials.

To improve the efficiency of the HMEVs isolation process, it is advisable to remove the potentially interfering milk components (such as somatic cells, milk fat, and casein micelles) by adding a preliminary purification step before the main HMEVs isolation process.

There are several methods for isolating HMEVs, which can be broadly categorized into density-based, size-based, and affinity-based techniques. Density-based isolation methods, such as differential ultracentrifugation and density gradient ultracentrifugation, separate HMEVs based on their density. Differential ultracentrifugation is considered the “gold standard” but is time-consuming and requires specialized equipment. Density gradient ultracentrifugation allows for more precise separation of HMEVs. Size-based isolation methods include polymer precipitation, membrane filtration, electrophoretic filtration, size exclusion chromatography, and microfluidics. Polymer precipitation using PEG and membrane filtration methods is simple and fast. Electrophoretic filtration uses an electric field to separate HMEVs based on their charge and size. Size exclusion chromatography employs porous gel columns, and microfluidics utilizes microscale systems for precise control of sample flow and separation. Affinity-based isolation methods, such as immunoisolation, use antibodies specific to HMEV surface proteins to selectively isolate vesicles. This method is highly specific but requires the availability of specific antibodies and can be costly. Additionally, commercial kits are available for HMEV isolation, offering convenience and speed, though their effectiveness depends on adherence to manufacturer protocols and specific research purposes.

Characterizing HMEVs involves various techniques to analyze their size, morphology, and molecular composition: FC allows for the analysis of HMEVs’ size, shape, and complexity. Enhanced flow cytometers can detect smaller particles, and fluorescence-based detection improves signal separation. DLS measures particle size distribution based on light scattering induced by Brownian motion. It is straightforward but less effective for heterogeneous samples. NTA estimates particle size and concentration by analyzing the diffusion rate of particles in a solution. It is widely used but can have high statistical uncertainties. TRPS measures particle size and quantity by detecting changes in electrical resistance as particles pass through a microporous membrane. It is precise but can be affected by pore blockage. EM, including SEM and TEM, provides high-resolution images of HMEVs’ surface and internal structures. Cryo-TEM preserves vesicle integrity for accurate size characterization. AFM offers high-resolution imaging of HMEVs’ morphology and size in their natural conditions with minimal sample preparation. Confocal Microscopy uses fluorescent dyes to image HMEVs, allowing for high-resolution analysis of their distribution and interactions with cellular components. WB detects specific proteins in HMEVs, confirming their presence and assessing sample purity.ELISA quantitatively measures specific proteins in HMEVs, providing high specificity for detecting subgroups like exosomes. LFIA offers rapid assessment of specific biomarkers in HMEVs, though not yet applied to mammalian milk EVs. By employing these characterization methods, researchers can gain comprehensive insights into the properties and functions of HMEVs, advancing our understanding of their roles in neonatal health and development.

Future research should focus on standardizing isolation and characterization methods for HMEVs to ensure reproducibility and comparability across studies. The development of consensus protocols for HMEV should standardize isolation methods, including centrifugation parameters (speed, time, temperature). Characterization protocols must also be optimized.

Platforms for sharing HMEV research data should be established, promoting transparency and collaboration among researchers. Multiple research groups should collaborate to develop highly characterized reference standards for HMEVs, covering size, protein markers, and RNA content. Workshops, training programs, and online resources (e.g., video tutorials and protocol databases) should support researchers in implementing standardized methods. By implementing these consensus protocols, the research community can enhance the reliability, reproducibility, and comparability of HMEV studies, advancing the field significantly.

Investigating the specific molecular mechanisms by which HMEVs influence infant development and immune responses will provide deeper insights into their functional roles. Additionally, exploring the impact of maternal health conditions on the composition and function of HMEVs could lead to personalized nutritional interventions. Longitudinal studies tracking the effects of HMEVs on infant health outcomes over time would be valuable in understanding their long-term benefits. Finally, developing advanced technologies for the targeted delivery of HMEVs could enhance their therapeutic potential in neonatal care.

## Author contributions

Conceptualization (KT, IKM, DMZ, equal contribution), Writing original draft (KT (lead), IKM (supporting), DMZ (supporting)), Writing - review and editing (KT, IKM, DMZ, equal contribution).

## Funding

The authors reported no funding received for this study.

## Conflict of interest

The authors report no conflicts of interest.
